# Complete Genome Sequences of Six Novel Macaca mulatta Papillomavirus Types Isolated from Genital Sites of Rhesus Monkeys in Hong Kong SAR, China

**DOI:** 10.1128/MRA.01414-18

**Published:** 2018-12-06

**Authors:** Teng Long, Po Yee Wong, Wendy C. S. Ho, Robert D. Burk, Paul K. S. Chan, Zigui Chen

**Affiliations:** a Department of Microbiology, Faculty of Medicine, The Chinese University of Hong Kong, Hong Kong Special Administrative Region, China; b Department of Pediatrics, Albert Einstein College of Medicine, Bronx, New York, USA; c Department of Microbiology and Immunology, Albert Einstein College of Medicine, Bronx, New York, USA; d Department of Epidemiology and Population Health, Albert Einstein College of Medicine, Bronx, New York, USA; e Department of Gynecology and Woman’s Health, Albert Einstein College of Medicine, Bronx, New York, USA; f Centre for Gut Microbiota Research, Faculty of Medicine, The Chinese University of Hong Kong, Hong Kong Special Administrative Region, China; University of Maryland School of Medicine

## Abstract

The complete genomes of six Macaca mulatta papillomavirus types isolated from genital sites of rhesus monkeys were characterized, and less than 72% identity with the complete L1 genes of known papillomaviruses was found. Macaca mulatta papillomavirus type 2 (MmPV2), MmPV3, and MmPV6 cluster into the genus *Alphapapillomavirus*, and MmPV4, MmPV5, and MmPV7 cluster into the genus *Gammapapillomavirus*.

## ANNOUNCEMENT

Papillomaviruses (PVs) are a heterogeneous family of viruses with circular double-stranded DNA genomes about 8 kb long that infect the epithelial surfaces of skin and oral and anogenital sites from a large spectrum of vertebrates, including reptiles, fish, birds, mammals, and humans. Persistent infection by high-risk human papillomaviruses (HPVs) has been linked to cervical cancer. PV genomes usually contain 6 early genes (E6, E7, E1, E2, E4, and E5), 2 late genes (L2 and L1), and an upstream regulatory region (URR) between the L1 and E6 genes. A distinct PV type is defined when the L1 nucleotide sequence is at least 10% dissimilar from that of any other type (1, 2). Currently, the complete genomes for more than 200 types of HPVs and 160 types of nonhuman animal PVs have been characterized (3).

In this report, we characterize the complete genomes of six novel Macaca mulatta PV types that are distributed in Hong Kong SAR, China, and were isolated from exfoliated vaginal cells of one adult female rhesus monkey and swabs from the penile surfaces of two adult male monkeys (Table 1). Total DNA was purified using a QIAamp DNA minikit (Qiagen, USA) and was preenriched for circular viral DNA using a TempliPhi amplification kit (GE Healthcare, USA). Next-generation sequencing libraries prepared with a KAPA HTP library preparation kit (Roche, USA) underwent metagenomic sequencing on a HiSeq 4000 platform (Illumina, USA) using paired-end 100-bp reads. A total of 92,908,115 raw sequence reads were trimmed for adaptors and low-quality bases using Trimmomatic v0.38 (4) and were further filtered for host genome contamination using Bowtie 2 v2.3.4 (5) against a rhesus chromosome reference (rheMac8, University of California Santa Cruz [UCSC]). High-quality short reads were assembled *de novo* to build contigs using MEGAHIT v1.1.3 (6). Whole viral genomes were validated using type-specific PCR amplification in three or four overlapping fragments and Sanger sequencing using a primer-walking strategy. Open reading frames (ORFs) were predicted using Geneious v9 and searched for homology using a BLASTX search against the closely related HPV types.

**TABLE 1:** Novel papillomaviruses isolated from rhesus monkeys (Macaca mulatta)

Macaca mulatta papillomavirus type	Isolate	Sex	Collection site	NCBI accession no.	Genus	Length in bp (% GC content)	Closest HPV type (L1 nt identity [%])^*a*^
2	PM069S3_c168082	Female	Vagina	MG837557	*Alphapapillomavirus*	7,876 (50.4)	HPV54 (70.7)
3	PM084S3_c177403	Male	Penis	MG837558	*Alphapapillomavirus*	8,051 (52.0)	HPV117 (70.3)
4	PM084S3_c176982	Male	Penis	MG837559	*Gammapapillomavirus*	7,424 (46.2)	HPV130 (65.7)
5	PM019S3_c169203	Male	Penis	MH745747	*Gammapapillomavirus*	7,408 (40.6)	HPV134 (71.7)
6	PM084S3_c170482	Male	Penis	MH745748	*Alphapapillomavirus*	7,935 (51.8)	HPV54 (71.6)
7	PM084S3_c160986	Male	Penis	MH745749	*Gammapapillomavirus*	7,292 (38.7)	HPVw34c04a (72.7)^*b*^

a nt, nucleotide.

b NCBI accession number MF588693.

Six novel PV genomes were named Macaca mulatta papillomaviruses 2, 3, 4, 5, 6, and 7 (MmPV2 to MmPV7, respectively) following the previously reported MmPV1 (7). The L1 nucleotide sequences of these novel PV types share 55.8% to 75.6% pairwise identity with each other and are less than 72% identical to those of any other PVs. A RAxML phylogenetic tree inferred from the L1 nucleotide sequences clustered MmPV2, -3, and -6 into the genus *Alphapapillomavirus*, and MmPV4, -5, and -7 cluster into the genus *Gammapapillomavirus*. Identification of novel papillomaviruses from nonhuman primate animals provides important insights into the complex phylodynamic interactions between viruses and primate hosts.

### Data availability.

The complete genome sequences of MmPV2 to MmPV7 were submitted to NCBI/GenBank under the accession numbers MG837557 to MG837559 and MH745747 to MH745749. The raw Illumina reads were deposited in the Sequence Read Archive under the accession numbers SRX4994337, SRX4994338, and SRX4994339. The phylogenetic tree was deposited in figshare (https://doi.org/10.6084/m9.figshare.7301432).
